# Spontaneous Colo-Umbilical Fistula Complicating Diverticulitis of the Sigmoid Colon

**DOI:** 10.1155/2013/925041

**Published:** 2013-06-06

**Authors:** Helen Bolanaki, Georgios Kouklakis, Nikos Courcoutsakis, Panagoula Oikonomou, Anastasios J. Karayiannakis

**Affiliations:** ^1^Second Department of Surgery, Democritus University of Thrace, Medical School, 68 100 Alexandroupolis, Greece; ^2^Endoscopy Unit, Democritus University of Thrace, Medical School, 68 100 Alexandroupolis, Greece; ^3^Department of Radiology and Medical Imaging, Democritus University of Thrace, Medical School, 68 100 Alexandroupolis, Greece

## Abstract

Colocutaneous fistula caused by diverticulitis is relatively uncommon with colo-umbilical fistulas being even rarer. We herein report a rare case of a spontaneous colo-umbilical fistula due to diverticulitis of the sigmoid colon. The fistula developed from a diverticulum of the sigmoid colon that discharged through the umbilicus after two episodes of acute diverticulitis. The condition was successfully treated by resectional surgery.

## 1. Introduction

Diverticular disease is an increasingly common condition in Western countries especially among elderly people. The disease affects mostly the left colon. It is usually asymptomatic and becomes manifest only when complicated with bleeding or inflammation. Acute diverticulitis may complicate with free perforation resulting in fecal or purulent peritonitis or with pericolic abscess formation, involvement of adjacent organs, and fistulation. Fistulas occur in approximately 5% of cases either spontaneously or after surgical or drainage procedures [1, 2]. The most common diverticulitis-associated fistulas are colovesical, colovaginal, and enterocolic, whereas colocutaneous fistulation has been only occasionally described [3–8]. We herein report a rare case of a spontaneous colo-umbilical fistula due to diverticulitis of the sigmoid colon. 

## 2. Case Presentation

A 54-year-old man presented with a 15-day history of low-grade fever and continuous mild pain around the umbilicus. Three days before admission he noticed foul smelling discharge emanating from the umbilical pit. His medical history included irritable bowel syndrome, diverticulosis of the sigmoid colon, and two episodes of acute diverticulitis over the last two years that were treated conservatively.

**Figure 1:**
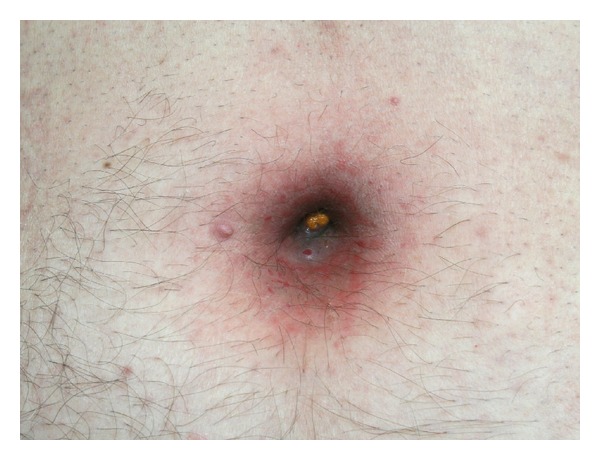
The inflamed and macerated umbilicus and periumbilical skin with fecal material into the umbilical pit.

On physical examination the abdomen was slightly distended with mild tenderness in the left lower quadrant without palpable masses or any evidence of acute abdomen. Guarding, rigidity, and rebound tenderness were absent and bowel peristalsis was normal. The umbilicus and the periumbilical skin were inflamed and macerated, with spontaneous fecal discharge from the umbilical pit (Figure 1). The laboratory results showed slightly elevated white blood cell counts (11,670/mm^3^) and C-reactive protein (2.8 mg/dL). A plain abdominal radiography was unremarkable.

**Figure 2:**
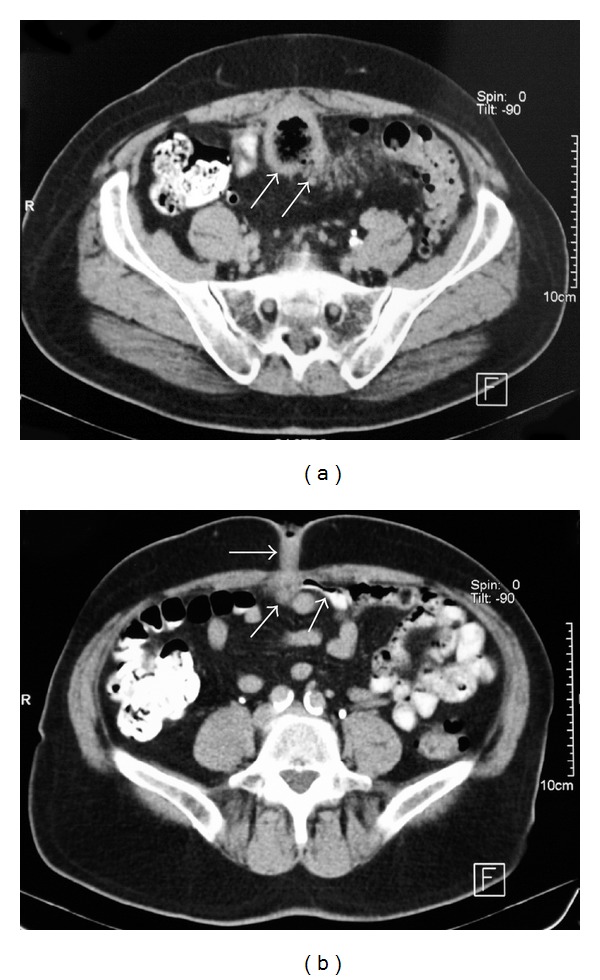
(a) Axial CT scan of the abdomen demonstrating diverticulosis of the sigmoid colon with inflammatory changes in the colonic wall (arrows) and mesenteric stranding with the sigmoid adherent to the posterior aspect of the anterior abdominal wall. (b) A thick-walled cavity (arrows) is present beneath the umbilicus communicating with the sigmoid colon.

Abdominal computed tomography (CT) revealed diffuse diverticulosis in the sigmoid colon with inflammatory changes and mesenteric stranding and a cavity beneath the abdominal wall at the level of the umbilicus communicating with the sigmoid colon (Figure 2). There were no signs of free perforation or free fluid in the abdomen. Colonoscopy revealed multiple diverticula but was negative for stenosis or tumor. Having enough imaging information a preoperative fistulography was considered redundant and was not performed.

**Figure 3:**
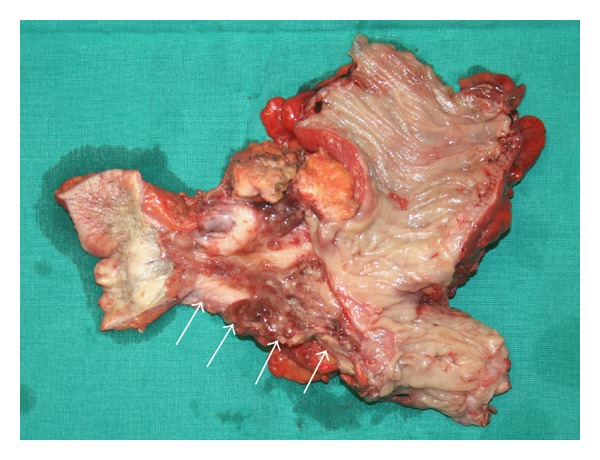
The resected specimen demonstrating the fibrotic fistulous tract (arrows) between the umbilicus and the sigmoid colon lined with granulation tissue.

Laparotomy demonstrated a fibrotic fistulous tract between the umbilicus and the sigmoid colon lined with granulation tissue attributable to the previous attacks of diverticulitis. The affected part of the sigmoid colon along with the fistulous tract and the umbilicus were resected en bloc (Figure 3), and a Hartmann's procedure was performed. A primary anastomosis was not considered due to moderate local inflammation consisting mainly of edema in the colonic wall and the pericolonic tissues and mesentery. Because of inflammatory and fibrotic changes in the area of intervention reported by the preoperative CT scan, the laparoscopic approach was considered difficult and was not employed. The pathology report confirmed diffuse colonic diverticular disease with signs of acute inflammation and an enterocutaneous fistula. Colostomy reversal with an end to end colorectal anastomosis was performed eight weeks later.

## 3. Discussion

Colonic fistulas to internal viscera such as urinary bladder, vagina, uterus, fallopian tube, ureter, jejunum, and colon or to the skin of the abdominal wall and the perineum (colocutaneous fistulas) arise either spontaneously due to perforation of a diverticular abscess into an adjacent organ or to the skin or as a complication after resection surgery for diverticulitis. Colovesical fistula is the most common internal fistula (48%) while colocutaneous fistula is relatively rare (5%) with the colo-umbilical type being even rarer. A colocutaneous fistula occurs usually after surgical intervention or percutaneous drainage of a diverticular abscess whilst spontaneous fistulation is uncommon [3–8]. In our patient the fistula probably resulted from perforation of a diverticulum at the umbilicus where the sigmoid colon was adherent following previous attacks of acute diverticulitis thus preventing generalized peritonitis.

The diagnosis of colocutaneous fistula is usually apparent from the history and the presence of a fistulous opening in the skin. Drainage of feces from the cutaneous opening is a constant and characteristic feature. Barium enema examination will often confirm the diagnosis. However, computed tomography is the preferred study for colocutaneous fistulas providing information on both the intra- and extraluminal features of the fistulous tract, the part of bowel involved, and the presence of abscesses, obstruction, or perforation [9]. Water soluble contrast fistulography outlines the fistulous tract and can demonstrate multiple fistulas. Colonoscopy is useful in excluding other pathologies.

Conservative management of colocutaneous fistulas consisting of withdrawal of oral intake, fluid and electrolyte replacement and nutritional support with total parental nutrition, control of both local and systemic sepses, and local skin care results in low closure rates and has the possibility of fistula recurrence. However, it can be used as an initial step to control local or systemic sepsis, to reduce the inflammation, and to lessen the operation. In our patient, the short and wide fistulous tact made spontaneous closure of the fistula very unlikely and thus surgical management was undertaken. 

Currently, resection of the fistulous tract and the affected colon with primary colonic anastomosis in a single stage is the treatment of choice for diverticulitis-associated fistulas. The success rate of the single-stage procedure is approximately 90% without increased postoperative morbidity and mortality rates [1, 2, 5]. Two- or three-stage procedures consisting of a preliminary diverting colostomy with or without resection, respectively, followed by colostomy closure as a final stage are a reasonable alternative especially in patients with massive fecal load, severe inflammation, or obstruction. More recently, the laparoscopic approach has been reported to be an effective and safe method for the management of diverticular disease complicated by fistulae [10, 11].

## 4. Conclusion

In conclusion, we here presented a rare case of spontaneous colo-umbilical fistula complicating diverticulitis of the sigmoid colon which was successfully treated by resectional surgery.
